# Monitoring the elimination of human African trypanosomiasis: Update to 2014

**DOI:** 10.1371/journal.pntd.0005585

**Published:** 2017-05-22

**Authors:** José R. Franco, Giuliano Cecchi, Gerardo Priotto, Massimo Paone, Abdoulaye Diarra, Lise Grout, Raffaele C. Mattioli, Daniel Argaw

**Affiliations:** 1 World Health Organization, Control of Neglected Tropical Diseases, Innovative and Intensified Disease Management, Geneva, Switzerland; 2 Food and Agriculture Organization of the United Nations, Sub-regional Office for Eastern Africa, Addis Ababa, Ethiopia; 3 Food and Agriculture Organization of the United Nations, Animal Production and Health Division, Rome, Italy; 4 World Health Organization, Regional Office for Africa, Communicable Disease Unit, Brazzaville, Congo; Institute of Tropical Medicine, BELGIUM

## Abstract

**Background:**

The World Health Organization (WHO) has targeted the elimination of Human African trypanosomiasis (HAT) ‘as a public health problem’ by 2020. The selected indicators of elimination should be monitored every two years, and we provide here a comprehensive update to 2014. The monitoring system is underpinned by the Atlas of HAT.

**Results:**

With 3,797 reported cases in 2014, the corresponding milestone (5,000 cases) was surpassed, and the 2020 global target of ‘fewer than 2,000 reported cases per year’ seems within reach. The areas where HAT is still a public health problem (i.e. > 1 HAT reported case per 10,000 people per year) have halved in less than a decade, and in 2014 they corresponded to 350 thousand km^2^. The number and potential coverage of fixed health facilities offering diagnosis and treatment for HAT has expanded, and approximately 1,000 are now operating in 23 endemic countries. The observed trends are supported by sustained surveillance and improved reporting.

**Discussion:**

HAT elimination appears to be on track. For gambiense HAT, still accounting for the vast majority of reported cases, progress continues unabated in a context of sustained intensity of screening activities. For rhodesiense HAT, a slow-down was observed in the last few years. Looking beyond the 2020 target, innovative tools and approaches will be increasingly needed. Coordination, through the WHO network for HAT elimination, will remain crucial to overcome the foreseeable and unforeseeable challenges that an elimination process will inevitably pose.

## Author summary

Human African trypanosomiasis (HAT), also known as sleeping sickness, is a neglected tropical disease transmitted by tsetse flies, which has been responsible for devastating epidemics in the 20^th^ century. Since the last alarming spike in disease incidence during the late 1990s, disease surveillance and control have been greatly strengthened, tremendous improvements have been achieved, and the disease is now targeted for elimination by the World Health Organization (WHO). In this paper we provide a comprehensive update of the indicators of HAT elimination to 2014, including number of reported cases and the areas and populations at risk. Fixed health facilities offering diagnosis and treatment for HAT were also surveyed, mapped, and their potential coverage of populations at risk was estimated. With 3,797 reported cases in 2014, the 2020 global target of ‘elimination as a public health problem’ (i.e. ‘fewer than 2,000 reported cases per year’) seems within reach. The 2030 target (i.e. elimination of transmission) is expected to pose more severe challenges. The sustained commitment of all stakeholders and close coordination of activities will have to be ensured, if the goal of HAT elimination is to be achieved.

## Introduction

In the last decade of the 20^th^ century, the number of cases of human African trypanosomiasis (HAT), also known as sleeping sickness, reached alarming levels [1,2,3,4]. In reaction to this epidemiological situation of a lethal disease, a number of stakeholders came together to support the affected countries. In the early years of the 21^st^ century, the World Health Organization (WHO) launched a public-private partnership that, together with important efforts from bilateral cooperation and non-governmental organizations (NGOs), enabled to reverse the epidemiological trend [5]. In this process, the key role was played by the National Sleeping Sickness Control Programmes (NSSCPs) of endemic countries and their committed health workers.

The steady reduction in the number of HAT cases reported during the first decade of the current century prompted first the HAT focal points of endemic countries [6], then the WHO Strategic and Technical Advisory Group on Neglected Tropical Diseases (NTDs) [7] and finally the WHO Expert Committee on control and surveillance of HAT [8] to set the elimination of HAT as a goal. The technical viability of HAT elimination rests on the existence of vulnerable points in the transmission cycle, the present as well as prospective availability of control tools, and the evidence of having reached elimination in several HAT transmission areas [9,10,11].

As a consequence, HAT was included in the WHO NTD roadmap as one of the diseases targeted for elimination as a public health problem by 2020 [7]. The indicators to measure the progress towards elimination were defined, and a reporting calendar was established [8]. The selected indicators for HAT elimination should be monitored every two years, and be presented in HAT stakeholders meetings [12].

A first progress report on HAT elimination looked at the gambiense form only [13], and it provided an update to 2012 for the main indicators of elimination. HAT elimination was shown to be on track, even though the exclusion of rhodesiense HAT data rendered that progress report incomplete. The present paper provides the first comprehensive biennial update, including both the gambiense and the rhodesiense form, and it covers the progress made in HAT elimination from 2000 to 2014. Reported data on HAT occurrence are compared to the milestones set by the WHO roadmap on NTDs [7], in particular as it concerns the target of fewer than 2,000 reported cases by 2020, which is the first global indicator of HAT elimination as a public health problem. Regarding the second global indicator (i.e. ‘number of foci reporting less than 1 case per 10,000 inhabitants’), we present here a revised metric based on the concept of ‘areas at risk of HAT’ [14], which enables a more robust and objective quantification. This revised metric was recently endorsed by the WHO HAT elimination Technical Advisory Group.

## Materials and methods

### Ethics statement

The research does not directly involve human participants. No individual data is used in the paper. All the data used are provided routinely by National Control Programmes as epidemiological information and are fully anonymized.

### Number of HAT cases reported annually

Detection of HAT cases is currently undertaken by NSSCPs, NGOs and Research Institutions. HAT morbidity data in disease-endemic countries are collected by NSSCPs or dedicated departments in the Ministries of Health, and subsequently reported to WHO on an annual basis. Field activities including active and passive case finding are regularly reported. Transboundary cases (i.e. individuals who contracted the infection in one country but who were detected by the health facilities in a neighbouring country) are also reported and allocated to the country of infection; national authorities are informed accordingly for appropriate action. Sporadic cases are also detected in non-endemic countries, amongst travellers and migrants. They are reported to WHO thanks to the centralized distribution of anti-trypanosome drugs. Information on the likely area of infection is used to allocate these ‘exported’ cases to the country of infection [15]. All data are entered in the database of the Atlas of HAT [16].

In the present paper, the number of HAT cases reported from 2000 to 2014 is provided for all endemic countries. These figures include a few minor revisions as compared to previously published counts for the period 2000–2013 [8,13,16,17]. The revisions stem from in-depth verifications carried out for the continuous improvement of the Atlas of HAT (e.g. a more accurate allocation of transboundary cases).

### Geographic distribution of HAT

The geographic distribution of HAT reported cases is mapped at the village level following already described methodologies [16,18]. The database includes, from the year 2000 onwards, not only the cases detected actively and passively but also the people examined per village during active screening activities carried out by mobile teams. All records in the database are linked to the source files from which the information was derived, and all source files are safely stored in a digital data repository.

In this paper, emphasis is given to the distribution of HAT cases for the five-year period 2010–2014. Because of the inherent epidemiological features of HAT, and in the context of the elimination strategy, a five-year window is considered as the most useful to analyse and present the updated picture of the extent of the disease [8,13]. In particular, the 5 year window is believed to strike a good balance between temporal resolution (which would call for a shorter window) and robustness (which would call for a longer window, so as to smooth the year-to-year variations in screening intensity).

### Areas and population at risk of HAT

The risk of HAT infection is estimated from the number of reported cases (numerator—Atlas of HAT) and the exposed population (denominator—Landscan [19]). Previously published methods enable point level data from the Atlas of HAT (village-level mapping) to be converted into continuous, smoothed surfaces of disease intensity and risk [14,20]. Smoothing is based on a 30-km radius kernel density, and although HAT risk was initially estimated over ten-year periods [14,20], more recently five-year periods have been considered more informative to monitor elimination [13]. In the present paper, the progress over time was investigated through a five-year sliding window (i.e. from 2000–2004 to 2010–2014). The 30-km smoothing for the estimation of HAT risk is meant to account for a variety of complex and not easily quantifiable epidemiological features such as the mobility of people and of the vector, whilst at the same time reducing the effect of mapping inaccuracies in the input data. This methodology and its rationale are described in detail elsewhere [14].

On the basis of the number of HAT cases per annum (p.a.) as compared to the exposed population, HAT risk is ranked into five categories: very high (≥ 1 HAT case per 10^2^ people), high (≥ 1 HAT case per 10^3^ people and < 1 per 10^2^ people), moderate (≥ 1 per 10^4^ people and < 1 per 10^3^ people), low (≥ 1 per 10^5^ people and < 1 per 10^4^ people), and very low (≥ 1 per 10^6^ people and < 1 per 10^5^ people) [13]. Risk is considered ‘marginal’ below the threshold of 1 HAT case p.a. per 10^6^ people. It is noteworthy that below the category of ‘moderate’, the risk level fits the WHO general definition of elimination of HAT as a public health problem.

For the present risk estimates, the Atlas of HAT provided village-level mapping for 92.9% of HAT reported cases (period 2000–2014). For the 7.1% of the cases which were not mapped at the village-level, information on the area of occurrence was used (i.e. unmapped cases were proportionally allocated to the endemic villages of the same area [20]).

### Population at risk potentially covered by fixed health facilities with capacities for HAT diagnosis and treatment

Fixed health facilities play a crucial role in the control and surveillance of HAT. With a view to estimating their physical accessibility and potential coverage of at-risk populations, time-distance analysis was used [21]. In the present paper the coverage of the population at risk of gambiense HAT is updated, and that of rhodesiense HAT is presented for the first time.

Data on the fixed health facilities that are active in HAT control and surveillance were provided by NSSCPs through standardized forms. For each health structure, information was collected on the name, location and capacities for HAT diagnosis and treatment. Data were harmonized, mapped and assembled in a geo-spatial database [21]. The survey was conducted between September 2015 and April 2016.

Diagnostic capacities for gambiense HAT were categorized as ‘clinical’ (DxC), ‘serological’ (DxS), ‘parasitological’ (DxP), and ‘stage determination’ (DxPh) [21]. For rhodesiense HAT, a serological screening test is not available, but the other three categories do apply. For treatment capacities, gambiense HAT includes treatment of infections in the first-stage, i.e. pentamidine (Tx1P), and in the second-stage, i.e. nifurtimox-eflornithine combination therapy—NECT (Tx2N), eflornithine (Tx2E) and melarsoprol (Tx2M) [21]. For rhodesiense HAT, treatment of first-stage infections with suramin (Tx1S) and of second-stage infections with melarsoprol (Tx2M) are available.

A time-distance function was used to map the physical accessibility to HAT diagnosis and treatment [21]. A ‘landscape friction’ geospatial layer for Africa provided the travel time through each 1-km/30 arcseconds pixel [22]. Landscape friction takes into account terrain and transportation network. The cumulative travel time was calculated from any location to the nearest health facility (‘shortest weighted distance’ or ‘least cumulated time’). The economic cost of travel (i.e. affordability) is not considered in our analysis; only travel time is computed.

For presentation purposes, results were summarized for three thresholds of travel time (i.e. ‘one hour’, ‘three hours’ and ‘five hours’) and stratified by risk categories. With a view to exploring trends, results for gambiense HAT are compared to those of a previous survey (completed in August 2013 [21]). For the previous study [21], stratification relied on a 10-year risk layer (2000–2009). To ensure consistency with the present estimates, which are stratified on a 5-year risk layer (2010–2014), previous estimates were recalculated on the basis of the corresponding 5-year risk layer (i.e. 2007–2011).

## Results

### Number of HAT cases reported annually

**Fig 1:**
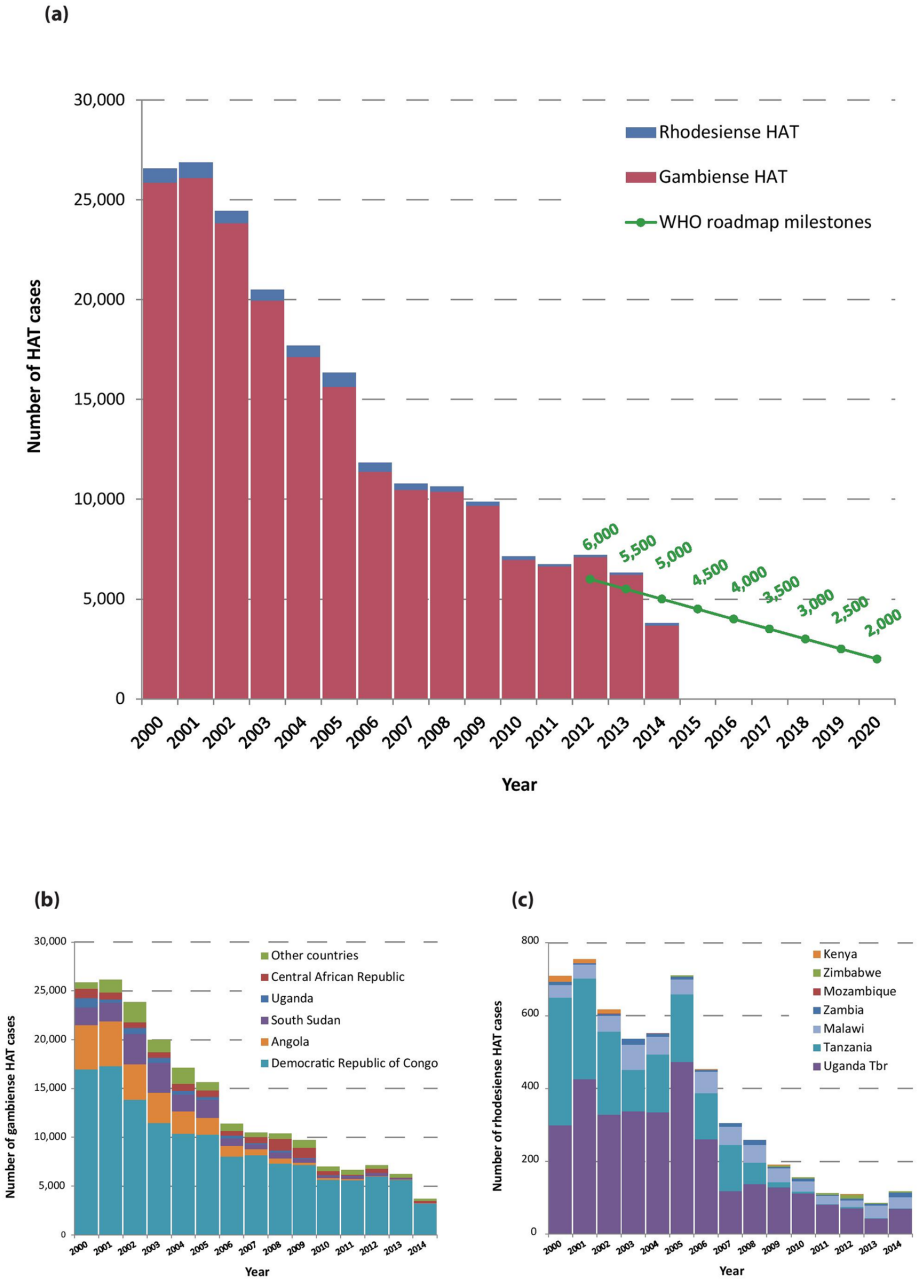
(a) Total number of reported cases of HAT (gambiense and rhodesiense) per year. The green line shows the milestones set in the WHO Roadmap for HAT elimination [7]. (b) Number of reported cases of gambiense HAT per year and per country. (c) Number of reported cases of rhodesiense HAT per year and per country.

A total of 3,797 new HAT cases (including both gambiense and rhodesiense HAT) were reported in 2014 (Fig 1). For this indicator, the continental target set for ‘HAT elimination as a public health problem’ is fewer than 2,000 cases, a level that was planned to be reached by the year 2020 [7]. The intermediate milestone of 5,000 cases in 2014 was not only reached but surpassed by 1,203 cases.

The number of gambiense HAT cases reported by year and by country is shown in Table 1. In 2014 a total of 3,679 cases were reported, corresponding to an 86% reduction compared to 2000. It is worth noting that the Democratic Republic of the Congo (DRC) continues to account for the vast majority of gambiense HAT cases. In 2014, the DRC accounted for 87% of the total number of cases (3,205 out of 3,679).

**Table 1:** *T*. *b*. *gambiense* HAT: New cases reported between 2000 and 2014.

Country	2000	2001	2002	2003	2004	2005	2006	2007	2008	2009	2010	2011	2012	2013	2014	Total
Angola	4,546	4,577	3,621	3,115	2,280	1,727	1,105	648	517	247	211	154	70	69	36	22,923
Cameroon	27	14	32	33	17	3	15	7	13	24	16	15	7	6	7	236
Central African Republic	988	718	572	539	738	666	460	654	1,194	1,054	395	132	381	59	194	8,744
Chad	153	138	715	222	483	190	276	97	196	510	232	276	197	195	95	3,975
Congo	111	894	1,005	717	873	398	300	189	182	87	87	61	39	20	21	4,984
Côte d’Ivoire	188	92	97	68	74	42	29	13	14	8	8	10	9	7	6	665
Democratic Republic of the Congo	16,951	17,300	13,816	11,459	10,339	10,249	8,013	8,155	7,318	7,178	5,624	5,590	5,968	5,647	3,205	136,812
Equatorial Guinea	16	17	32	23	22	17	13	15	11	7	8	1	2	3	0	187
Gabon	45	30	26	26	49	53	31	30	24	14	22	17	9	17	10	403
Ghana	1	0	0	0	0	0	0	0	0	0	0	0	0	1	0	2
Guinea	52	72	132	130	95	94	48	69	90	79	68	57	70	78	33	1,167
Nigeria	14	14	26	31	10	21	3	0	0	0	2	3	2	0	0	126
South Sudan	1,801	1,919	3,121	3,061	1,742	1,853	789	469	623	373	199	272	317	117	63	16,719
Uganda	948	310	604	517	378	311	290	120	198	99	101	44	20	9	9	3,958
Total	25,841	26,095	23,799	19,941	17,100	15,624	11,372	10,466	10,380	9,680	6,973	6,632	7,091	6,228	3,679	200,901

Other *T*. *b*. *gambiense* HAT endemic countries not reporting cases but with surveillance activities are Benin, Burkina Faso, Mali, Niger, Senegal, Sierra Leone, and Togo. In Gambia, Guinea-Bissau and Liberia no cases are reported but no surveillance activity is known.

**Fig 2:**
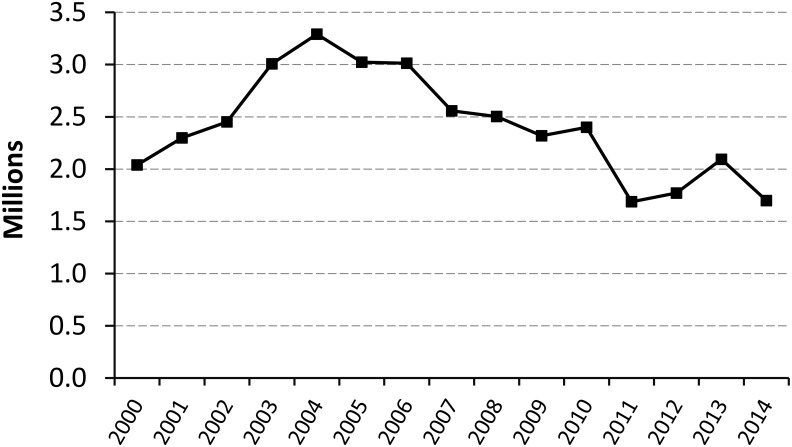
Number of people screened by active case-finding surveys, in countries endemic for *T*. *b*. *gambiense* (2000–2014).

Fig 2 shows the number of people screened by active case-finding surveys in countries endemic for *T*. *b*. *gambiense* in the period 2000–2014. The chart shows that, despite year-to-year variations, the overall intensity of active surveillance has been fairly stable over the described time period, and it has stabilized at approximately 1.8 million people screened per year over the last four years (2011–2014).

Concerning rhodesiense HAT, results are shown in Table 2. With 118 cases reported in 2014, the rhodesiense form of the disease continues to represent a small part of the total HAT reported cases (3%). With 709 cases reported in the year 2000, a reduction of 83% in 14 years was observed. Over the last 4 years, the number of rhodesiense HAT cases has stabilized at around 100 per year.

**Table 2:** *T*. *b*. *rhodesiense* HAT: New cases reported between 2000 and 2014.

Country	2000	2001	2002	2003	2004	2005	2006	2007	2008	2009	2010	2011	2012	2013	2014	Total
Kenya	15	10	11	0	0	0	1	0	0	1	0	0	2	0	0	40
Malawi	35	38	43	70	48	41	58	50	49	39	29	23	18	35	32	608
Mozambique	-	-	1	-	1	-	-	-	-	-	-	-	-	-	-	2
Uganda	300	426	327	338	335	473	261	119	138	129	112	84	71	43	70	3,226
United Republic of Tanzania	350	277	229	113	159	185	127	126	59	14	5	1	4	1	1	1,651
Zambia	9	4	5	15	9	7	6	10	13	4	8	3	6	6	12	117
Zimbabwe	-	-	-	-	-	3	-	-	0	3	2	4	9	1	3	25
Total	709	755	616	536	552	709	453	305	259	190	156	115	110	86	118	5,669

Other *T*. *b*. *rhodesiense* HAT endemic countries not reporting cases are Burundi, Ethiopia and Rwanda. Botswana, Namibia and Swaziland are considered free of the vector for the transmission of *T*. *b*. *rhodesiense* HAT [16].

### Geographic distribution of HAT

**Fig 3:**
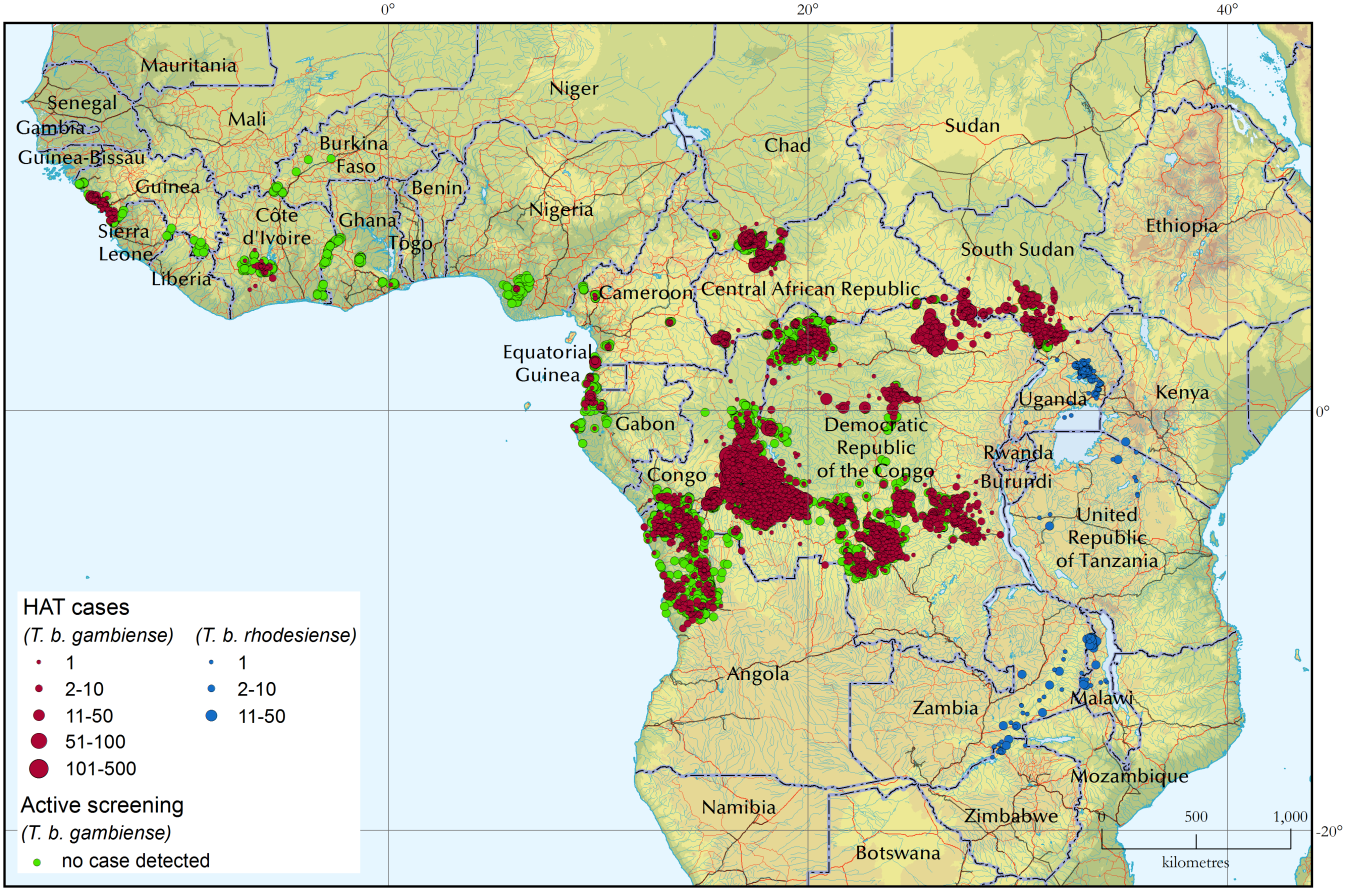
The distribution of human African trypanosomiasis. Period 2010–2014. Red circles (gambiense HAT cases) are plotted so as to overlay green circles (active screening campaigns in which no HAT case was detected). As a result, only the green circles that are at the fringes of gambiense HAT distribution are visible.

Fig 3 shows the geographic distribution of sleeping sickness cases for the 5-year period 2010–2014. The locations of active screening activities where no cases were detected are also included (green circles). For the period 2010–2014, 31,188 new HAT cases were reported, 88.3% of which could be mapped at the village level.

For the whole period 2000–2014 (2000 being the start year of the Atlas of HAT), a total number of 206,570 cases has been included in the database. Of these, 92.9% cases have been mapped at the village level, for a total of 30,278 mapped villages. The average accuracy for mapped HAT cases is presently estimated at 1.3 km, and it is being continuously improved.

#### Gambiense HAT

In West Africa, disease transmission continues in Guinea. The decrease in reported cases in 2014 can be ascribed to the abrupt reduction in case-finding activities during the Ebola outbreak, during which only weak passive case detection was in place. In Côte d'Ivoire, the number of recorded cases keeps decreasing even following a reinforcement of active and passive case detection. In Nigeria, cases are only sporadically reported, against a backdrop of insufficient case detection activities. One autochthonous case was detected by passive surveillance in Ghana in 2013, after over 10 years without any reported infection. The subsequent reactive active screening in the area (Shai Osudoku District, Greater Accra Region) detected no additional case. In Benin, Burkina Faso, Ghana, Mali and Togo sentinel sites have been established for passive surveillance integrated in general health facilities. According to NSSCP, in these five countries, a total of 1,108 HAT clinically suspected individuals were screened in the period 2013–2014 in the sentinel sites, but no HAT case was diagnosed.

In Central Africa, active screening activities are routinely carried out in Cameroon, Chad, Congo, Equatorial Guinea and Gabon, and the system of passive case detection has been reinforced. In these countries, the decreasing trend in the number of cases continues and it is believed to reflect a real abatement in disease transmission (5,720 cases in 2000–2004, 2,702 in 2005–2009 and 1,363 in 2010–2014). Cases have also been recently reported from an old transmission area in Southern Chad (Maro). In Central African Republic the number of reported cases continues to decrease, but the data from this country have to be interpreted with care; in fact, active screening activities have been erratic and seriously hampered by insecurity in the Prefectures of Haut Mbomou and Ouham.

In Uganda the number of gambiense HAT cases has dropped dramatically (from 1,018 in 2005–2009 to 183 in the following five year period). As of 2013, the substantial reinforcement of passive detection in the endemic areas gives a high confidence in a real decline in HAT transmission. In South Sudan, HAT reported cases decreased sharply, from 4,107 in 2005–2009 to 968 cases in 2010–2014. However, these figures need to be interpreted with caution because of the diminishing intensity of screening and surveillance activities [23].

In Angola, HAT reported cases continued to decrease steadily (from 4,244 in 2005–2009 to 540 in 2010–2014). This trend was observed in a context where capacities for passive detection remained in place, but active case-finding activities were significantly scaled down.

The DRC remains the country with the heaviest burden of HAT, but the number of cases continues to decrease (from 69,865 in 2000–2004 to 40,913 in 2005–2009 and to 26,034 in 2010–2014). The reported trend is likely to reflect a real reduction of infections, as the intensity of active and passive surveillance remained remarkably stable over these years, with approximately 2 million people screened per year. Interestingly, vector control activities were recently initiated in a few foci of gambiense HAT, with a view to complementing medical activities. Vector control is therefore believed to have contributed to the observed positive trends in such countries as Guinea [24] and Chad.

#### Rhodesiense HAT

The number of rhodesiense HAT reported cases has been decreasing steadily in the majority of affected countries (Table 2). Uganda reported 65% of all rhodesiense HAT cases during the period 2010–2014, even while experiencing a reduction of 66% as compared with the previous 5 years (from 1,120 cases in 2005–2009 to 380 in 2010–2014). In Uganda, although a few cases are reported from protected areas probably due to the wildlife reservoir, the main reservoir involved in transmission is cattle [25]. Multisectoral disease control activities including the veterinary dimension were reinforced over the years in a One Health framework, thus enabling disease transmission to be brought under control [26]. A very substantial reduction was observed also in Tanzania, from 511 reported cases in 2005–2009 to 12 in 2010–2014, although in the meantime the capacities for surveillance have weakened. In Malawi, reported cases decreased from 237 in 2005–2009 to 137 in 2010–2014, but progress has stagnated over the last few years. In Zambia the epidemiological situation appears stationary, with an average of eight cases per year (42 in 2000–2004, 40 in 2005–2009 and 35 in 2010–2014). In Zimbabwe, the Zambezi Valley is the area of disease transmission; no cases were reported in 2000–2004, while 6 and 19 cases were detected in 2005–2009 and 2010–2014 respectively [27]. Despite the increase in reported cases, it is difficult to establish whether disease transmission is on the increase in Zimbabwe, as in the period 2000–2004 the reporting system was weaker than in following years.

### Areas and population at risk of HAT

#### Areas at risk of HAT

**Fig 4:**
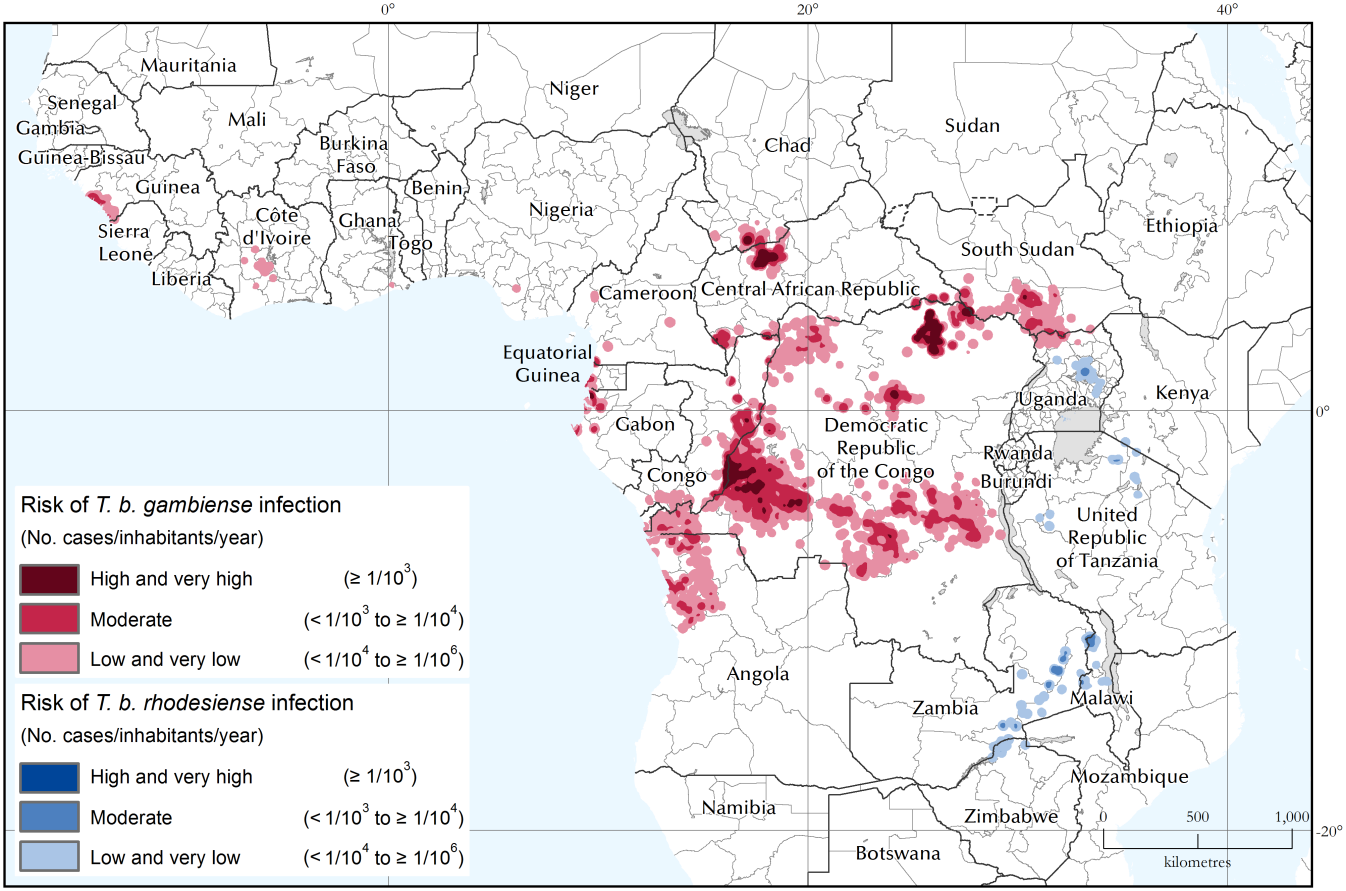
The areas at risk of HAT infection. Period 2010–2014.

**Fig 5:**
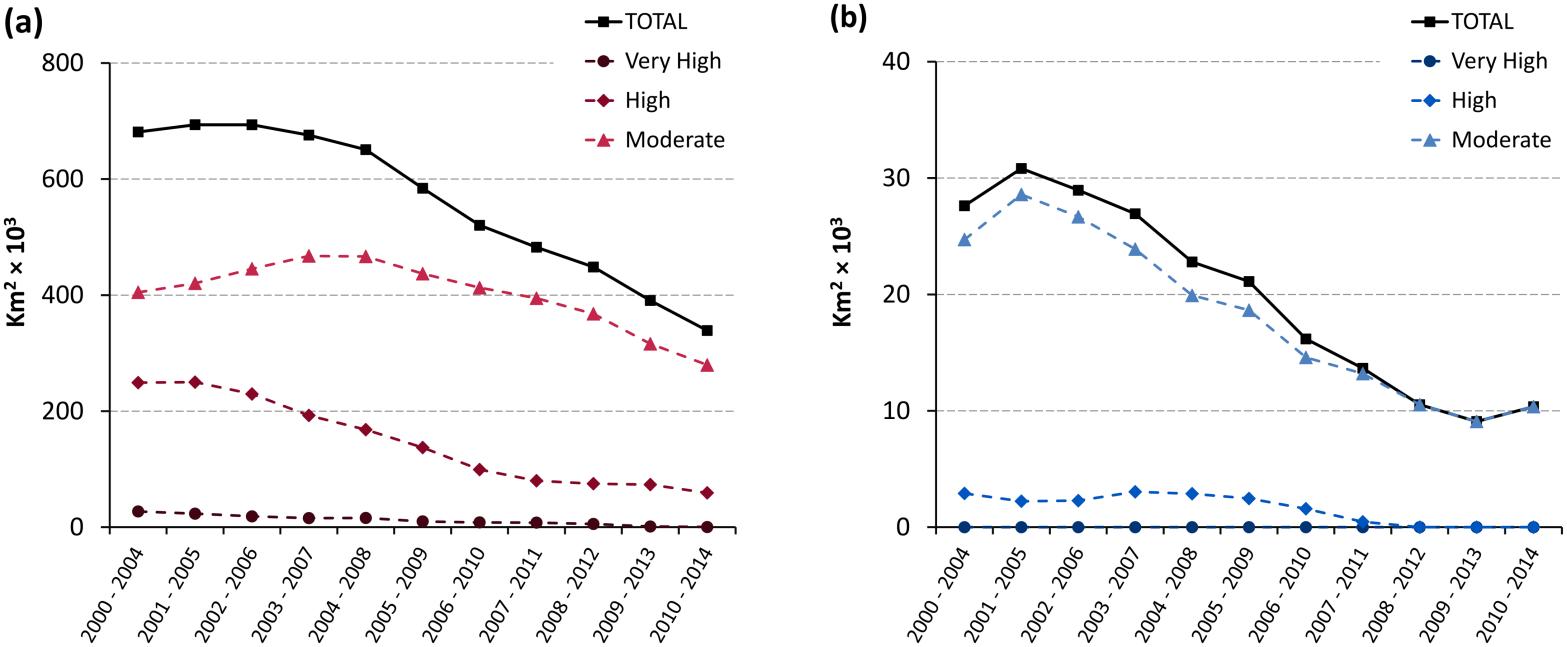
Trends in areas at risk of gambiense HAT (a) and rhodesiense HAT (b) from 2000–2004 to 2010–2014.

Fig 4 shows the areas at risk of HAT for the 5-year period 2010–2014. The areas are also summarized by country in Tables 3 and 4 for gambiense and rhodesiense HAT respectively. In the two tables the 2010–2014 figures are compared with the period 2005–2009. Lastly, Fig 5 shows the evolution of the areas at HAT risk between 2000–2004 and 2010–2014.

**Table 3:** Areas at risk of *T*. *b*. *gambiense* infection (km^2^). Periods 2005–2009 and 2010–2014.

Country	Total country area*	Area at risk 2005–2009					Area at risk 2010–2014				
		Very High and High	Moderate	Low and Very Low	Total at risk	% of total country area	Very High and High	Moderate	Low and Very Low	Total at risk	% of total country area
Angola	1,253,770	19,443	66,349	80,094	165,885	13.2	-	18,128	85,979	104,107	8.3
Cameroon	466,396	-	1,181	16,156	17,337	3.7	33	1,603	8,685	10,320	2.2
Central African Republic	624,398	20,383	19,282	21,331	60,996	9.8	8,161	20,338	28,258	56,757	9.1
Chad	1,272,490	3,108	3,992	7,138	14,238	1.1	1,740	4,142	16,138	22,021	1.7
Congo	338,522	14,800	23,517	37,312	75,629	22.3	3,768	21,620	44,687	70,075	20.7
Côte d’Ivoire	321,363	-	138	18,561	18,700	5.8	-	-	12,042	12,042	3.7
Democratic Republic of the Congo	2,304,080	70,833	260,162	389,614	720,609	31.3	44,043	186,528	445,571	676,142	29.3
Equatorial Guinea	27,019	-	3,314	2,611	5,925	21.9	-	1,376	2,793	4,169	15.4
Gabon	265,978	1,663	4,912	5,524	12,099	4.5	625	4,761	8,813	14,198	5.3
Ghana	234,325	-	-	-	-	-	-	-	738	738	0.3
Guinea	246,094	-	3,754	9,621	13,375	5.4	25	2,825	8,970	11,819	4.8
Nigeria	908,866	-	-	3,469	3,469	0.4	-	-	1,662	1,662	0.2
Sierra Leone	72,777	-	-	1,147	1,147	1.6	-	-	1,118	1,118	1.5
South Sudan	633,356	16,387	44,276	37,120	97,783	15.4	1,076	17,091	64,300	82,468	13.0
Uganda	205,540	645	6,076	9,535	16,255	7.9	-	1,128	12,220	13,348	6.5
Other Endemic Countries**	3,228,725	-	-	-	-	-	-	-	-	-	-
Total	12,403,699	147,262	436,953	639,233	1,223,447	9.9	59,470	279,539	741,974	1,080,983	8.7

* Land area. The area of surface water bodies as depicted in the Shuttle Radar Topography Mission—River-Surface Water Bodies dataset is not included.

** Countries at marginal risk: Benin, Burkina Faso, Gambia, Guinea-Bissau, Liberia, Mali, Niger, Senegal and Togo.

**Table 4:** Areas at risk of *T*. *b*. *rhodesiense* infection (km^2^). Periods 2005–2009 and 2010–2014.

Country	Total country area*	Area at risk 2005–2009					Area at risk 2010–2014				
		Very High and High	Moderate	Low and Very Low	Total at risk	% of total country area	Very High and High	Moderate	Low and Very Low	Total at risk	% of total country area
Burundi	25,053	-	-	151	151	0.6	-	-	-	-	-
Kenya	574,883	-	-	969	969	0.2	-	-	2,362	2,362	0.4
Malawi	94,758	179.848	3,105	10,428	13,712	14.5	-	2,380	10,108	12,488	13.2
Mozambique	779,061	-	-	-	-	-	-	-	482	482	0.1
United Republic of Tanzania	886,278	2,251	9,310	33,067	44,628	5.0	-	303	15,436	15,739	1.8
Uganda	205,540	-	3,316	25,740	29,056	14.1	-	1,602	17,340	18,942	9.2
Zambia	742,479	32.9259	2,914	35,807	38,754	5.2	-	6,071	36,749	42,820	5.8
Zimbabwe	388,414	-	-	7,610	7,610	2.0	-	6	8,681	8,687	2.2
Other Endemic Countries**	2,568,511	-	-	-	-	-	-	-	-	-	-
Total	6,264,977	2,464	18,644	113,773	134,880	2.2	-	10,362	91,158	101,520	1.6

* Land area. The area of surface water bodies as depicted in the Shuttle Radar Topography Mission—River-Surface Water Bodies dataset is not included.

** Countries at marginal risk: Botswana, Ethiopia, Namibia, Rwanda and Swaziland.

For gambiense HAT, 1.08 million square kilometres are estimated to be at risk of infection (period 2010–2014), with almost sixty thousand square kilometres at very high and high risk, and almost two hundred and eighty thousand at moderate risk. Over 62% of the total area at risk is in the DRC. Compared to the previous 5 years (2005–2009), the total area at risk decreased by 12% (Table 3), with more marked declines for the categories at higher risk (i.e. 60% and 36% for ‘high and very high’ and ‘moderate’ respectively). Areas at risk shrank in virtually all endemic countries, with the exception of Chad (+55%), and Gabon (+17%). In absolute terms, the most sizable reductions were observed in Angola (-62,000 km2) and DRC (-44,000 km2).

Fig 5a focuses on the cumulated categories of moderate, high and very high risk. These categories correspond to the areas where HAT is considered a public health problem (i.e. > 1 HAT case p.a. per 10,000 people). Also, at the global level, they correspond to the second primary indicator to monitor the elimination process, i.e. ‘number of foci reporting less than 1 case per 10,000 inhabitants’. We observe that the areas at moderate, high and very high risk halved from 2004 to 2014 (from approximately 680 thousand km^2^ to less than 340 thousand km^2^). The decrease was sharper in the higher risk categories, with the areas at high or very high risk shrinking by 78% (from 276 to 59 thousand km^2^). Looking at individual countries, the largest strides in reducing the areas where gambiense HAT is a public health problem were made in the DRC (with a reduction of approximately 125,000 km^2^ over the last 11 years), Angola (107,000 km^2^), South Sudan (45,000 km^2^) and Congo (40,000 km^2^).

For rhodesiense HAT, approximately 100,000 km^2^ are estimated to be at risk of infection (Table 4). Most of these areas are in the low and very low risk categories (92,000 km^2^), where HAT is not considered a public health problem. Only 10,000 km^2^ are at moderate risk, while no area affected by rhodesiense HAT was estimated to be at high or very high risk in the period 2010–2014. The trends over the past 11 years are presented in Fig 5b. We note that for rhodesiense HAT, in the first monitored period 2000–2004, the extent of the transmission areas where the disease can be considered a public health problem were already much smaller than for gambiense HAT (i.e. 27,600 km^2^ for rhodesiense HAT versus 681,000 km^2^ for gambiense HAT). Even if already starting from these relatively small areas, by 2010–2014 the reduction was even sharper in relative terms (i.e. 62% reduction for rhodesiense HAT as compared to 50% for gambiense HAT). Focusing on individual countries, the most sizable elimination of rhodesiense HAT as a public health problem was achieved in Tanzania, with a reduction of over 13,000 km^2^, followed by Uganda (almost 4,000 km^2^), although the reduction in Tanzania has to be interpreted in a context of considerably reduced surveillance activities.

#### Population at risk of HAT

For gambiense HAT, 55 million people are estimated to be at risk of infection (period 2010–2014), with 1.2 million at very high and high risk, and 9.2 million at moderate risk (Table 5). Therefore, over 10 million people live in areas where gambiense HAT is still considered a public health problem. Most of these people live in DRC (8.5 million). As compared to the previous 5 years (i.e. 2005–2009), the total population at risk increased by over 5 million, but the increase only concerns the low and very low risk categories (+11 million). General population growth and people changing status from higher to lower risk categories account for the growth in the population at low and very low risk, which in turn explains the growth in the total population at risk. People living in areas where gambiense HAT is still a public health problem (i.e. risk categories ‘very high’ to ‘moderate’), decreased by 6 million.

**Table 5:** Population at risk of *T*. *b*. *gambiense* infection (no. persons × 10^3^). Periods 2005–2009 and 2010–2014.

Country	Total country population 2009*	Population at risk 2005–2009					Total country population 2014*	Population at risk 2010–2014				
		Very High and High	Moderate	Low and Very Low	Total at risk	% of total country population		Very High and High	Moderate	Low and Very Low	Total at risk	% of total country population
Angola	12,799	260	859	1,386	2,504	19.6	19,088	-	304	3,996	4,300	22.5
Cameroon	18,879	-	15	473	488	2.6	23,131	-	25	197	221	1.0
Central African Republic	4,511	74	104	239	417	9.2	5,278	58	203	294	555	10.5
Chad	10,329	95	146	225	467	4.5	11,412	58	185	703	946	8.3
Congo	4,013	30	166	2,114	2,310	57.6	4,662	12	80	2,288	2,380	51.0
Côte d’Ivoire	20,617	-	38	1,666	1,704	8.3	22,849	-	-	1,300	1,300	5.7
Democratic Republic of the Congo	68,693	2,054	10,711	21,876	34,640	50.4	77,434	1,065	7,486	29,481	38,032	49.1
Equatorial Guinea	633	-	24	18	42	6.6	722	-	17	14	31	4.2
Gabon	1,515	7	21	767	794	52.4	1,673	3	19	856	878	52.5
Ghana	23,888	-	-	-	-	-	25,758	-	-	55	55	0.2
Guinea	10,058	-	170	2,180	2,351	23.4	11,474	-	146	1,133	1,279	11.1
Nigeria	149,229	-	-	721	721	0.5	177,156	-	-	468	468	0.3
Sierra Leone	5,132	-	-	128	128	2.5	5,744	-	-	117	117	2.0
South Sudan	9,054	189	617	452	1,259	13.9	11,563	13	681	1,703	2,397	20.7
Uganda	32,370	19	822	1,189	2,030	6.3	35,919	-	99	2,016	2,116	5.9
Other Endemic Countries**	79,785	-	-	-	-	-	91,146	-	-	-	-	-
Total	451,505	2,728	13,692	33,434	49,855	11.0	525,009	1,210	9,244	44,621	55,075	10.5

* As per Landscan

** Countries at marginal risk: Benin, Burkina Faso, Gambia, Guinea-Bissau, Liberia, Mali, Niger, Senegal and Togo.

**Fig 6:**
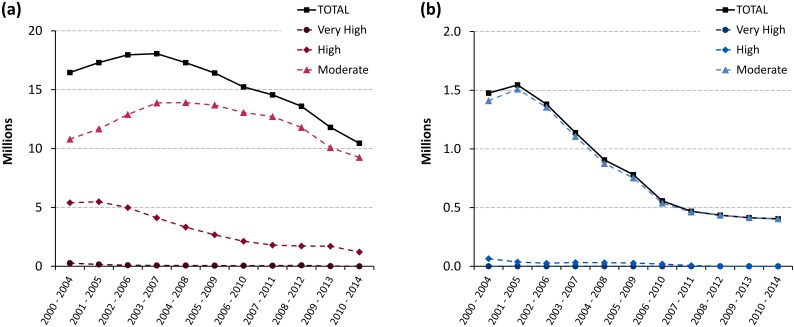
Trends in population at risk of gambiense HAT (a) and rhodesiense HAT (b) from 2000–2004 to 2010–2014.

Fig 6a shows the abatement in disease transmission at the continental level. The reduction in the three combined risk categories moderate, high and very high was 36% from 2000–2004 to 2010–2014 (from 16.5 to 10.5 million people). In relative terms, the most dramatic reduction was in the very high risk category (99%, from 265 thousand to 2 thousand people). For population at high and moderate risk the reductions were respectively 78% (from 5.4 to 1.2 million) and 14% (from 10.8 to 9.2 million).

For rhodesiense HAT, over 6 million people are estimated to be at risk (Table 6). Most are in the low and very low risk categories (5.6 million), while only four hundred thousand people are in the moderate risk category. In Fig 6b, we note that the reduction in the three combined risk categories for rhodesiense HAT was 73% (from 1.47 to 0.4 million people). The population at high risk decreased from 65 thousands to zero (2004 to 2014), while the population at very high risk was always zero for rhodesiense HAT throughout the monitored period.

**Table 6:** Population at risk of *T*. *b*. *rhodesiense* infection (no. persons × 10^3^). Periods 2005–2009 and 2010–2014.

Country	Total country population 2009*	Population at risk 2005–2009					Total country population 2014*	Population at risk 2010–2014				
		Very High and High	Moderate	Low and Very Low	Total at risk	% of total country population		Very High and High	Moderate	Low and Very Low	Total at risk	% of total country population
Burundi	9,511	-	-	17	17	0.2	10,396	-	-	-	-	-
Kenya	39,003	-	-	316	316	0.8	45,010	-	-	46	46	0.1
Malawi	15,029	-	96	843	939	6.2	17,377	-	82	754	837	4.8
Mozambique	21,669	-	-	-	-	-	24,692	-	-	4	4	0.0
United Republic of Tanzania	41,049	27	147	990	1,163	2.8	49,639	-	1	479	480	1.0
Uganda	32,370	-	497	7,145	7,642	23.6	35,919	-	304	3,654	3,958	11.0
Zambia	11,863	-	13	353	367	3.1	14,639	-	17	417	434	3.0
Zimbabwe	11,393	-	-	96	96	0.8	13,772	-	-	148	148	1.1
Other Endemic Countries**	101,420	-	-	-	-	-	114,744	-	-	-	-	-
Total	283,306	28	753	9,760	10,540	3.7	326,188	-	404	5,501	5,905	1.8

* As per Landscan

** Countries at marginal risk: Botswana, Ethiopia, Namibia, Rwanda and Swaziland.

### Population at risk potentially covered by fixed health facilities with capacities for HAT diagnosis and treatment

#### Survey and mapping of fixed health facilities

For gambiense HAT, the survey completed in February 2016 revealed the existence of 882 fixed health facilities with capacity for diagnosis (+28% as compared to the survey in 2013 [21]), out of which 516 also have capacity for treatment. Sixty-three percent of the 882 facilities are found in the DRC. The complete results of the surveys, and the comparison with the 2013 survey [21], are provided in Table 7.

**Table 7:** Fixed health facilities for gambiense HAT: Survey September 2015—February 2016 (columns ‘2016’), and differences to the survey December 2012 –August 2013 [21] (columns ‘Δ’).

Country	Diagnosis										Treatment										TOTAL	
	DxC		DxS		DxP		DxPh		Total Dx		Tx1P		Tx2M		Tx2E		Tx2N		Total Tx			
	2016	Δ	2016	Δ	2016	Δ	2016	Δ	2016	Δ	2016	Δ	2016	Δ	2016	Δ	2016	Δ	2016	Δ	2016	Δ
Angola	19	-	19	-	18	-	17	-	19	-	17	-	0	-12	14	+1	9	+9	17	-	19	-
Benin	3	-	3	-	0	-	0	-	3	-	0	-	0	-	0	-	0	-	0	-	3	-
Burkina Faso	7	+5	7	+5	2	-	1	-	7	+5	1	-	1	-	1	-	0	-	1	-	7	+5
Cameroon	11	+3	2	+2	8	+1	8	+1	11	+3	10	+1	0	-6	4	-1	6	+1	10	+1	11	+2
Central African Republic	16	+5	9	-	9	-	9	-	16	+5	10	-	8	-	7	-	7	-	10	-	16	+5
Chad	26	+22	24	+20	6	+2	6	+2	26	+22	26	+16	3	-1	6	+2	6	+2	26	+16	26	+16
Congo	10	-	10	-	6	-2	4	-3	10	-	5	-4	0	-5	3	-	3	-	5	-4	10	-
Côte d’Ivoire	4	-	4	-	1	-	1	-	4	-	4	-	1	-	1	-	1	-	4	-	4	-
Democratic Republic of the Congo	557	+33	348	+75	244	+15	195	+22	557	+33	410	+6	153	+6	144	-	173	+29	410	+6	557	+33
Equatorial Guinea	4	-1	4	+3	2	-2	1	-	4	-1	2	-2	1	-	1	-	1	-	2	-2	4	-1
Gabon	4	+3	4	+3	1	-	1	-	4	+3	1	-3	0	-2	1	-1	1	-1	1	-3	4	-
Ghana	8	+7	6	+6	0	-	0	-	8	+7	8	+7	0	-	0	-	0	-	8	+7	8	+7
Guinea	11	+8	11	+9	7	+5	3	+1	11	+8	3	+1	2	+1	0	-1	3	+2	3	+1	11	+8
Mali	11	+5	6	+5	1	-	1	-	11	+5	1	-	1	-	1	-	1	-	1	-	11	+5
Nigeria	50	+45	50	+45	5	-	5	-	50	+45	5	-	0	-5	0	-	5	+5	5	-	50	+45
South Sudan	15	+5	6	-	12	+5	6	-1	15	+5	9	-1	6	-1	6	-1	6	-1	9	-1	15	+5
Togo	2	-	2	-	0	-	0	-	2	-	0	-	0	-	0	-	0	-	0	-	2	-
Uganda	124	+120	124	+120	4	-	4	-	124	+120	4	-	4	-	4	-	4	-	4	-	124	+120
Total	882	+260	639	+293	326	+24	262	+22	882	+260	516	+21	180	-25	193	-1	226	+46	516	+21	882	+250

DxC: clinical diagnosis; DxS: serological diagnosis; DxP: parasitological diagnosis; DxPh: disease staging. Tx1P: treatment of first-stage infection with pentamidine; Tx2M: treatment of second-stage infection with melarsoprol; Tx2E: treatment of second-stage infection with eflornithine; Tx2N: treatment of second-stage infection with nifurtimox-eflornithine combination therapy (NECT); Tx2: treatment of second-stage.

While clinical suspects can be identified in all of the 882 facilities, serological testing is available in 72% of them (639, i.e. +46%). The more complex and advanced types of diagnosis, i.e. parasitological diagnosis and disease staging, are available in only 326 (+7%) and 262 (+8%) of the facilities respectively.

A more limited number of facilities offer treatment for gambiense HAT (516, +4%), all of which can administer pentamidine for first-stage infections. First line treatment for second-stage infections (NECT) is provided by 226 facilities (+20%), a relatively low number as this treatment requires highly skilled personnel for administration.

For rhodesiense HAT, 111 facilities offer diagnosis in six endemic countries, i.e. Kenya, Malawi, Uganda, United Republic of Tanzania, Zambia and Zimbabwe (Table 8). All of these perform clinical diagnosis, while parasitological diagnosis and disease staging are offered by 44% and 31% of the facilities respectively. Thirty-two health facilities are involved in rhodesiense HAT treatment, and all provide both suramin for first-stage infections and melarsoprol for second-stage.

**Table 8:** Fixed health facilities for rhodesiense HAT: survey 2016 (Survey September 2015—April 2016).

Country	Diagnosis				Treatment			TOTAL
	DxC	DxP	DxPh	Total Dx	Tx1S	Tx2M	Total Tx	
Kenya	25	1	1	25	1	1	1	25
Malawi	20	10	6	20	4	4	4	20
Uganda	38	14	7	38	7	7	7	38
United Republic of Tanzania	15	12	10	15	11	11	11	15
Zambia	12	11	9	12	8	8	8	12
Zimbabwe	1	1	1	1	1	1	1	1
Total	111	49	34	111	32	32	32	111

DxC: clinical diagnosis; DxP: parasitological diagnosis; DxPh: disease staging. Tx1S: treatment of first-stage infection with suramin; Tx2M: treatment of second-stage infection with melarsoprol.

**Fig 7:**
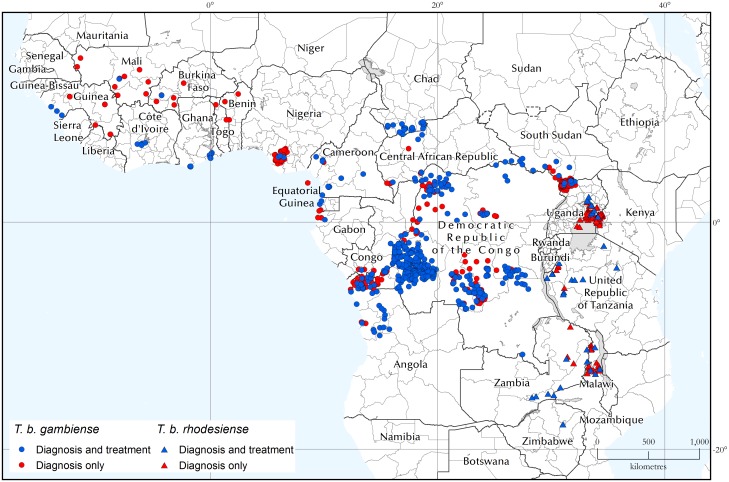
Geographic distribution of fixed health facilities offering diagnosis and treatment of gambiense and rhodesiense HAT. Data were collected by WHO from National Sleeping Sickness Control Programmes between September 2015 and April 2016.

The geographic distribution of the health structures involved in HAT diagnosis and treatment are shown in Fig 7. S1 Text provides maps of the centres able to offer the different types of diagnosis and treatment.

#### Population at risk potentially covered by fixed health facilities

Table 9 summarizes the potential coverage of the population at risk of gambiense HAT by fixed health facilities. For diagnosis, 28, 43, and 48 million people at risk are respectively within one, three and five hours travel of a competent facility (corresponding to 52%, 77%, and 88% of the at-risk population). For treatment, the corresponding figures are 23 (42%), 40 (73%), and 47 million (86%). These figures show an improved situation as compared to the previous survey, when accessibility for the same categories was 43%, 74% and 81% for diagnosis and 39%, 72% and 85% for treatment.

**Table 9:** People at risk of gambiense HAT that are potentially covered by facilities with diagnostic and treatment capabilities for HAT.

Risk category	People at risk	People at risk covered by facilities with HAT capabilities											
		Diagnosis						Treatment					
		≤ 1-hour travel		≤ 3-hour travel		≤ 5-hour travel		≤ 1-hour travel		≤ 3-hour travel		≤ 5-hour travel	
	(no. persons × 10^3^)	(no. persons × 10^3^)	% of at risk	(no. persons × 10^3^)	% of at risk	(no. persons × 10^3^)	% of at risk	(no. persons × 10^3^)	% of at risk	(no. persons × 10^3^)	% of at risk	(no. persons × 10^3^)	% of at risk
High and very high	1,210	549	45	1,004	83	1,116	92	538	44	1,000	83	1,115	92
Moderate	9,244	4,014	43	6,913	75	8,146	88	3,749	41	6,786	73	8,070	87
Low and very low	44,621	23,804	53	34,628	78	39,171	88	18,931	42	32,488	73	38,070	85
Total	55,075	28,367	52	42,546	77	48,432	88	23,219	42	40,274	73	47,255	86

When looking at the coverage by risk categories, clear improvements were observed in the very low, low and moderate risk, whilst comparatively lower gains were measured in the high and very high risk categories. It is noteworthy that an improvement in accessibility was observed for the more complex levels of diagnosis (serological, parasitological and disease staging) as well as treatment (second-stage infections) (S2 Text).

Among the population at risk of rhodesiense HAT (Table 10), 2.5, 4.6, and 5.2 million people are respectively within one, three and five hours travel of a diagnostic-competent facility (corresponding to 41%, 75%, and 86% of the at-risk population). For treatment, the corresponding estimates are 2.0, 4.4, and 5.1 million (i.e. 34%, 72%, and 84% of the at-risk population).

**Table 10:** People at risk of rhodesiense HAT that are potentially covered by facilities with diagnostic and treatment capabilities for HAT.

Risk category	People at risk	People at risk covered by facilities with HAT capabilities											
		Diagnosis						Treatment					
		≤ 1-hour travel		≤ 3-hour travel		≤ 5-hour travel		≤ 1-hour travel		≤ 3-hour travel		≤ 5-hour travel	
	(no. persons × 10^3^)	(no. persons × 10^3^)	% of at risk	(no. persons × 10^3^)	% of at risk	(no. persons × 10^3^)	% of at risk	(no. persons × 10^3^)	% of at risk	(no. persons × 10^3^)	% of at risk	(no. persons × 10^3^)	% of at risk
High and very high	0	-	-	-	-	-	-	-	-	-	-	-	-
Moderate	404	247	61	356	88	393	97	219	54	331	82	378	94
Low and very low	5,658	2,260	40	4,202	74	4,820	85	1,853	33	4,046	72	4,697	83
Total	6,062	2,507	41	4,557	75	5,213	86	2,071	34	4,377	72	5,075	84

Importantly, health facilities providing HAT diagnosis and treatment also cover a substantial number of people who are at marginal risk, including areas that were at risk of the disease in the past.

## Discussion

### Epidemiological trends

The data presented in this paper, covering the 15 years between 2000 and 2014, indicate clear progress towards HAT elimination as a public health problem, which is on track to be achieved by 2020. For the first time, in 2014 fewer cases than the set milestone were reported (i.e. 3,797 against the 5,000 milestone). The level reported in 2014 had been planned to be reached by 2016–2017. This decrease in reported cases was observed in a context of fairly constant intensity of active screening activities and reinforced passive surveillance in several countries, so the trend is very likely to reflect a real abatement in disease transmission. Preliminary data for 2015 (not presented here) show a further reduction in reported cases, thus corroborating the observed trend.

Areas at risk of HAT, which are estimated from reported cases and exposed population, are also shrinking. In particular, the areas where HAT is still a public health problem (i.e. where ≥ 1 HAT case per 10^4^ people p.a. is reported), have been decreasing steadily. Between 2004 and 2014 we estimate a reduction of approximately 360,000 km^2^, i.e. -51% as compared to the 2004 level.

The number of fixed facilities providing gambiense HAT diagnosis and treatment increased, and thus their potential coverage of the at-risk population. Improvements were observed in the basic levels of diagnosis (clinical, serological) and treatment (first-stage), as well as in the more advanced levels (i.e. parasitological diagnosis, disease staging, and first-line treatment for second-stage infection with NECT).

Looking at rhodesiense HAT, we note that this form of the disease continues to represent a relatively small proportion of the total number of HAT reported cases (i.e. 2.7% average for 2000–2014). Against this backdrop, a sizeable decrease was observed between 2000 and 2011, with a reduction of 594 cases p.a. (i.e. 84%). However, since 2011 progress has stagnated and the number of reported cases has stabilized at around 100 p.a. Several factors may have contributed to the stagnation. One is the expanded use of rapid diagnostic tests (RDT) for diagnosing malaria instead of microscopic examination. In fact, microscopic examination enabled the accidental diagnosis of HAT while looking for malaria parasites. Another is the fact that, in the first decade of 2000, the maximum reduction of cases was observed in areas where livestock is the reservoir of *T*. *b*. *rhodesiense*, and where strengthened veterinary public health brought about the decrease [26]. The wildlife reservoir is much more difficult to manage and it represents the main source of the scattered but constant rhodesiense HAT cases that are reported from protected areas [16]. It is also important to note that for rhodesiense HAT the rate of under-detection is likely to be higher than for gambiense HAT. The causes of this are manifold, and they include a faster disease progression, the poor effectiveness of active screening that makes rhodesiense HAT only detectable by passive screening, a lower incidence and therefore a lower awareness and preparedness of health staff, and the occurrence of the disease in sparsely populated areas. As a result of this likely higher rate of under-detection, the reliability of all indicators based on reported cases is bound to be lower for rhodesiense than for gambiense HAT.

Applying to rhodesiense HAT the same risk threshold as for gambiense HAT, we observe that the areas where the disease is still a public health problem are very few and very small. In the period 2010–2014 they were mostly limited to a restricted area in central Uganda (Kaberamaido and Dokolo Districts [28]) and the area surrounding the Vwaza Marsh Wildlife Reserve in Malawi [29]. A few additional scattered areas at moderate risk are also found in sparsely populated zones of Zambia (mostly corresponding to the Luangwa National Parks [30]).

Looking at the potential coverage of the at-risk populations by passive surveillance, we provide here the first continental survey of fixed facilities having capacity for diagnosis and treatment of rhodesiense HAT. At this stage, we only note that the coverage of population at risk of rhodesiense and gambiense HAT is comparable. One hundred and eleven facilities for rhodesiense HAT were identified, compared with 882 for gambiense HAT, which at first glance seems proportionate to the respective reported burdens. However, when looking at these figures, the substantial differences between the two diseases have to be kept in mind, and especially the generally lower transmission intensity and the likely higher under-detection for rhodesiense HAT. In this context, we argue that there is a need to expand and improve the network of fixed health facilities for rhodesiense HAT (e.g. in Zimbabwe [27]). In this analysis, financial barriers are not considered for both gambiense and rhodesiense HAT, but it is important to underline that in many countries, it can be an important barrier that prevent a wider use of the existing facilities.

### Methodological challenges in monitoring HAT elimination

In this paper, efforts were made to present a comprehensive update of the indicators of elimination, as set by WHO [8]. We show that, in the framework of the Atlas of HAT, data were assembled and methodologies were developed that enable us to follow the progress of HAT elimination with a high level of geographic detail (village-level mapping), and completeness of data in time and space (comprehensive data from all reporting countries are systematically collated, harmonized and analyzed from the year 2000 onwards). However, challenges in monitoring the process of elimination still remain.

Regarding the primary global indicators of elimination, while the cumulative number of HAT reported cases has been effectively followed by WHO for a long time, monitoring the second primary indicator, i.e. ‘the number of foci where HAT is no longer a public health problem’ [8], is proving more challenging. The main reason for this is that the available definition of focus as ‘a zone of transmission to which a geographical name is given (locality, region, or river)’ [31] is useful for operational purposes, but it is vague and not particularly helpful for measuring. In particular, it is difficult to define the geographical boundaries of HAT foci in an objective and standardized way. The challenge is compounded by our incomplete understanding of the focal nature of HAT [16], and by the fact that different countries use different criteria to define foci. On the other hand, the data collected in the HAT Atlas allow to quantify the area at risk [13,14], which is, in essence, a ‘zone of transmission’ that can be measured in a robust and objective way. As such, it represents a much more suitable metric for the second primary indicator of HAT elimination, originally proposed as ‘number of foci reporting less than 1 case per 10,000 inhabitants’. This metric also easily lends itself to monitoring over time, and at various spatial scales (from global to subnational and local levels). A recently created WHO HAT elimination Technical Advisory Group (HAT-e-TAG) recognized the impossibility of enumerating and delineating HAT foci objectively, and endorsed the revised global metric to assess elimination as a public health problem (i.e. the ‘total area at risk reporting ≥ 1 case /10,000 people/year’, which corresponds to the risk categories of ‘moderate’, ‘high’ and ‘very high’). HAT-e-TAG also proposed the 2020 target for this indicator, i.e. a reduction of 90% by the year 2020 as compared to the baseline calculated for the period 2000–2004.

Regarding the secondary indicators of elimination, they currently include population at risk, coverage of active and passive screening activities, and the geographic distribution of the disease [8].

For population at risk, the available data and methodologies enable an effective monitoring. Provided that attention is paid to the various levels of risk, the population at risk provides useful complementary information to the primary indicators.

Regarding the coverage of passive surveillance, at this stage we are in a position to estimate only a potential coverage (i.e. physical accessibility), and there is still a two-year lag between the survey of health facilities and the risk map used for stratification. In the future, efforts will be made to shorten the lag, and, much more importantly, to estimate the actual coverage of passive surveillance from the number of individuals passively screened by the health facilities. As to active screening activities, data are already systematically included in the Atlas of HAT that will enable the actual coverage to be estimated and mapped. To this end, a methodology is presently being developed. It is worth pointing out that the present methodologies to estimate coverage fail to capture issues of quality of coverage, such as what age, sex, or occupational groups are covered, quality and performance of the services provided and the varying efficacy of detection methods used.

As regards geographic distribution, the last secondary indicator, it is still considered as a very useful aspect of the epidemiology of HAT to be monitored. However, measuring this indicator quantitatively is not deemed particularly relevant at this stage, especially because the indicator ‘area at risk’ already captures the main quantitative aspects of the geographic distribution of HAT.

One cross-cutting aspect that affects virtually all indicators is their reliance on reported HAT cases, with all the uncertainties that an unknown level of underdiagnosis and underreporting brings. Efforts are being made to estimate and map these uncertainties through geospatial and environmental modelling [32,33].

### Conclusions

The advances in the process of HAT elimination [13] are confirmed in this new comprehensive report for gambiense and rhodesiense HAT. In particular, the milestone for the number of HAT cases reported in 2014 was achieved and even surpassed. Case-finding efforts were sustained in most of the affected countries, which gives confidence in a real progress in disease elimination. These results were accomplished mainly through sustained efforts in disease surveillance and control by NSSCPs.

The strength of the epidemiological knowledge continues to improve. The database of the Atlas of HAT is regularly improved in terms of completeness and accuracy, thus resulting in a robust estimation of the indicators. At the same time, models trying to predict the level of underdetection and the presence or absence of the disease in grey areas are being developed.

In a few affected areas, access to diagnosis and treatment is still constrained by insecurity (e.g. in Central Africa Republic and South Sudan) and remoteness (e.g. in some area in the DRC). Also, the progressive loss of expertise and motivation of health staff dealing with HAT is one of the inevitable effects of the reduction of cases. New innovative approaches are required to sustain the quality of interventions. Looking to the future, another inevitable consequence of reduced number of cases will be a progressive shift from active screening to a combination of passive surveillance and reactive screening. This shift, and the related integration of gambiense HAT surveillance into the health system, will be one of the main challenges to elimination.

Rhodesiense HAT represents a relatively small part of the global HAT problem. Because of its zoonotic dimension, the approach to tackle rhodesiense HAT must consider the epidemiological role of the domestic and wild animal reservoir in a One Health framework. As a result, disease elimination will require a multisectoral approach that should involve the veterinary services and include a vector control component [34,35]. As to the interruption of transmission, it is likely to remain elusive for some time to come, unless a breakthrough in control tools enables to tackle the animal reservoir (especially its wildlife compartment).

Despite the recent advances, it is crucially important to sustain the commitment of all stakeholders. Appropriate funding must be ensured if the 2020 and the 2030 goals are to be achieved. It is likely that both targets can be met, although the latter (i.e. interruption of transmission) is expected to pose a more severe challenge, and it is only applicable to gambiense HAT [8]. In the process of elimination, increased ownership of the fight against HAT by endemic countries must be ensured. The challenges to integration of HAT activities in weak national health systems raise concerns. All efforts and policies aiming to strengthen health systems, especially in rural areas, will contribute to the sustainability of HAT elimination.

Looking at gambiense HAT, in this new context of strongly reduced prevalence, human asymptomatic carriers [36] and the possible animal reservoir [37,38] need to be studied in more detail, as they could play a role in disease maintenance, resurgence and reintroduction. Development of new control tools, including diagnosis, treatment and vector control, could change the current control and surveillance scenario by enabling innovative, adapted and more cost-effective strategies to be implemented.

While the process of HAT elimination is progressing as planned, many challenges still lie ahead. At this juncture, the WHO network for HAT elimination set up in 2014 [12] ensures crucial coordination of stakeholders and maximum effectiveness in the support to endemic countries. Only by maintaining the synergy and coordination of interventions will sustainable elimination of HAT be achieved.

## Supporting information

S1 Text Geographic distribution of fixed health facilities having capacities for different types of diagnosis and treatment of human African trypanosomiasis. Fig A Geographic distribution of fixed health facilities having capacities for clinical diagnosis of gambiense and rhodesiense HAT (i) and serological diagnosis of gambiense HAT (ii) Fig B Geographic distribution of fixed health facilities having capacities for parasitological diagnosis of HAT (i) and stage determination (ii) Fig C Geographic distribution of fixed health facilities having capacities for treatment of gambiense HAT first-stage infections with pentamidine and of rhodesiense HAT first-stage infections with suramin (i) and second-stage infection with melarsoprol (ii) Fig D Geographic distribution of fixed health facilities having capacities for treatment of gambiense HAT second-stage infections with eflornithine (i) and with nifurtimox-eflornithine combination therapy (ii).
(DOCX)

S2 Text People at risk of HAT that are potentially covered by facilities with diagnostic and treatment capabilities for HAT Table A. People at risk of Gambiense HAT that are potentially covered by facilities with diagnostic capabilities (2016, 2013 and difference 2016–2013) Table B. People at risk of Gambiense HAT that are potentially covered by facilities with treatment capabilities (2016, 2013 and difference 2016–2013) Table C. People at risk of Rhodesiense HAT that are potentially covered by facilities with diagnostic capabilities (2016) Table D. People at risk of Rhodesiense HAT that are potentially covered by facilities with treatment capabilities (2016).
(DOCX)
