# Supplementary material for: Monitoring the elimination of human African trypanosomiasis: Update to 2014
Source: PLoS Negl Trop Dis. 2017 May 22;11(5):e0005585. doi: 10.1371/journal.pntd.0005585 (PMC5456402; doi:10.1371/journal.pntd.0005585)
Supplement: S1 Text — Fig A Geographic distribution of fixed health facilities having capacities for clinical diagnosis of gambiense and rhodesiense HAT (i) and serological diagnosis of gambiense HAT (ii) Fig B Geographic distribution of fixed health facilities having capacities for parasitological diagnosis of HAT (i) and stage determination (ii) Fig C Geographic distribution of fixed health facilities having capacities for treatment of gambiense HAT first-stage infections with pentamidine and of rhodesiense HAT first-stage infections with suramin (i) and second-stage infection with melarsoprol (ii) Fig D Geographic distribution of fixed health facilities having capacities for treatment of gambiense HAT second-stage infections with eflornithine (i) and with nifurtimox-eflornithine combination therapy (ii). (DOCX) [file pntd.0005585.s001.docx]

**Additional file**

**Geographic distribution of fixed health facilities having capacities for diagnosis of human African trypanosomiasis**
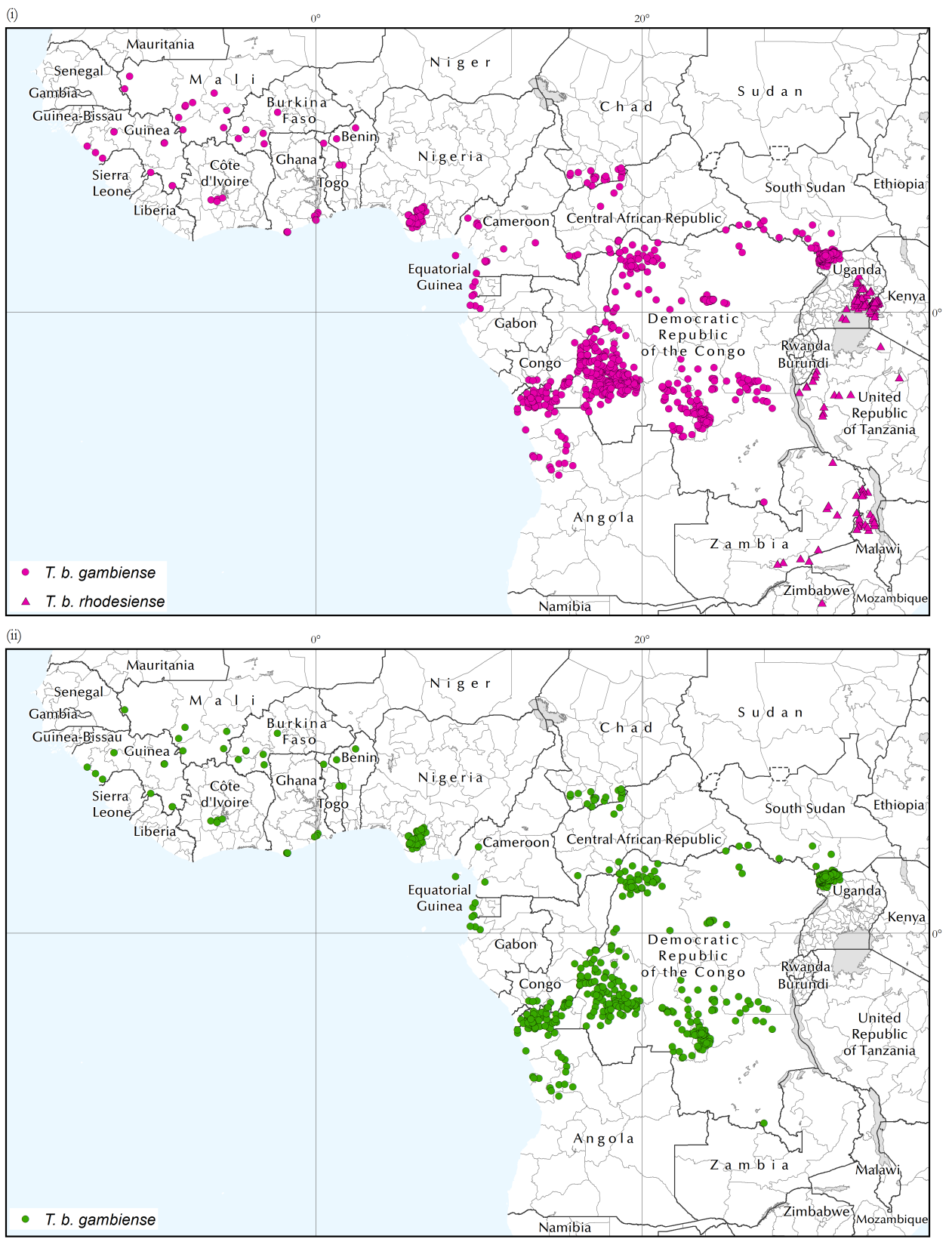


Fig A Geographic distribution of fixed health facilities having capacities for clinical diagnosis of gambiense and rhodesiense HAT (i) and serological diagnosis of gambiense HAT (ii)


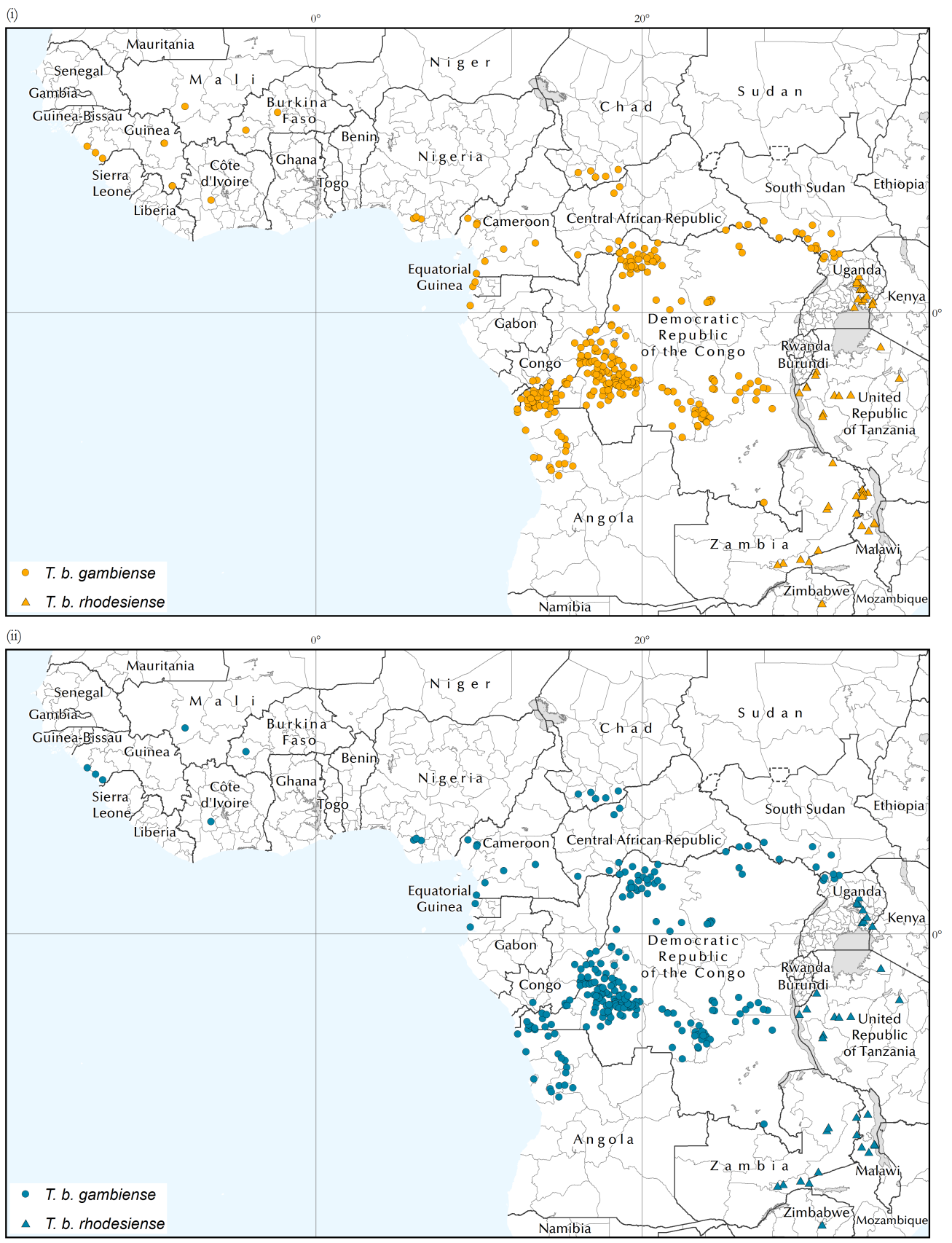


Fig B Geographic distribution of fixed health facilities having capacities for parasitological diagnosis of HAT (i) and stage determination (ii)

**Geographic distribution of fixed health facilities having capacities for treatment of human African trypanosomiasis**
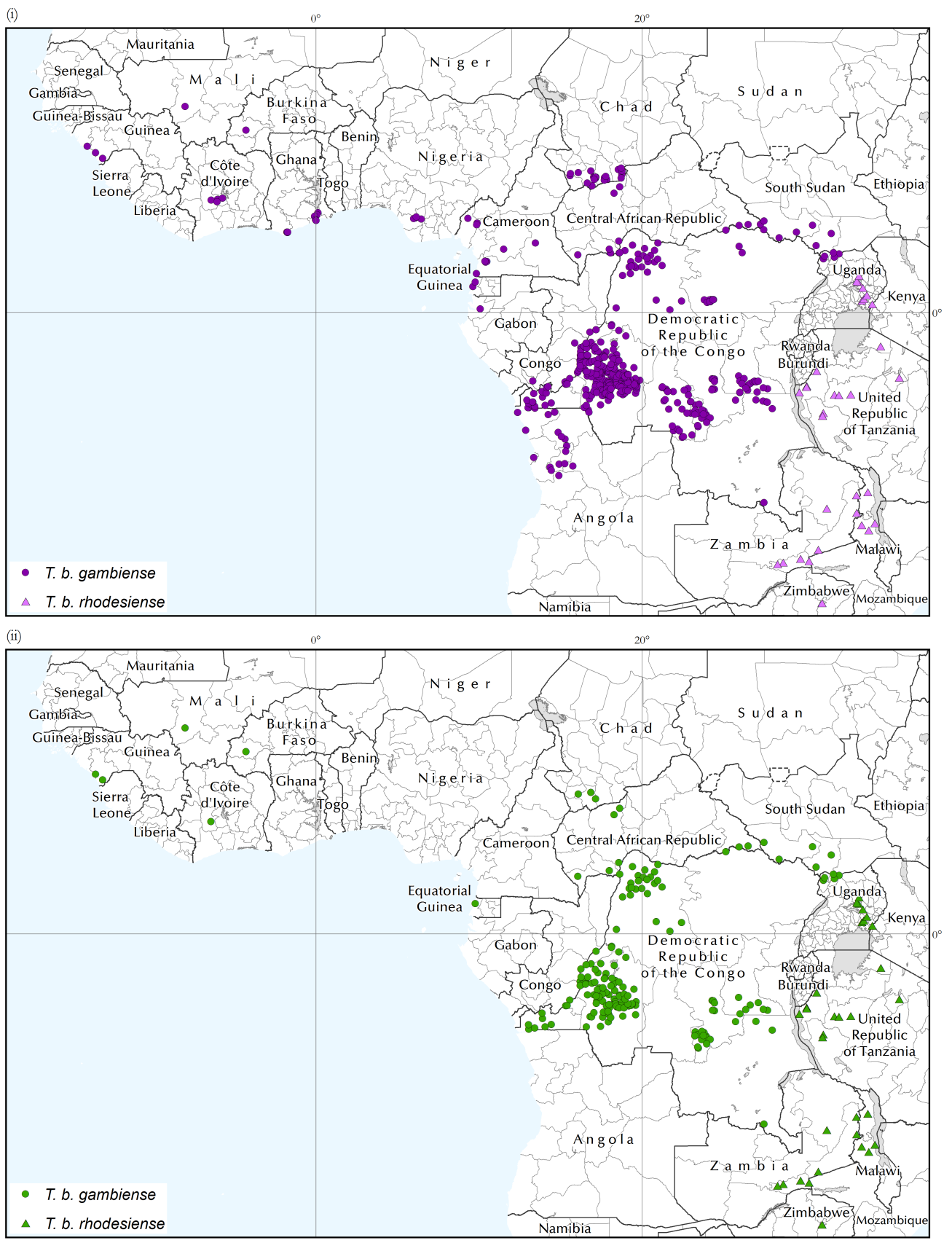


Fig C Geographic distribution of fixed health facilities having capacities for treatment of gambiense HAT first-stage infections with pentamidine and of rhodesiense HAT first-stage infections with suramin (i) and second-stage infection with melarsoprol (ii)


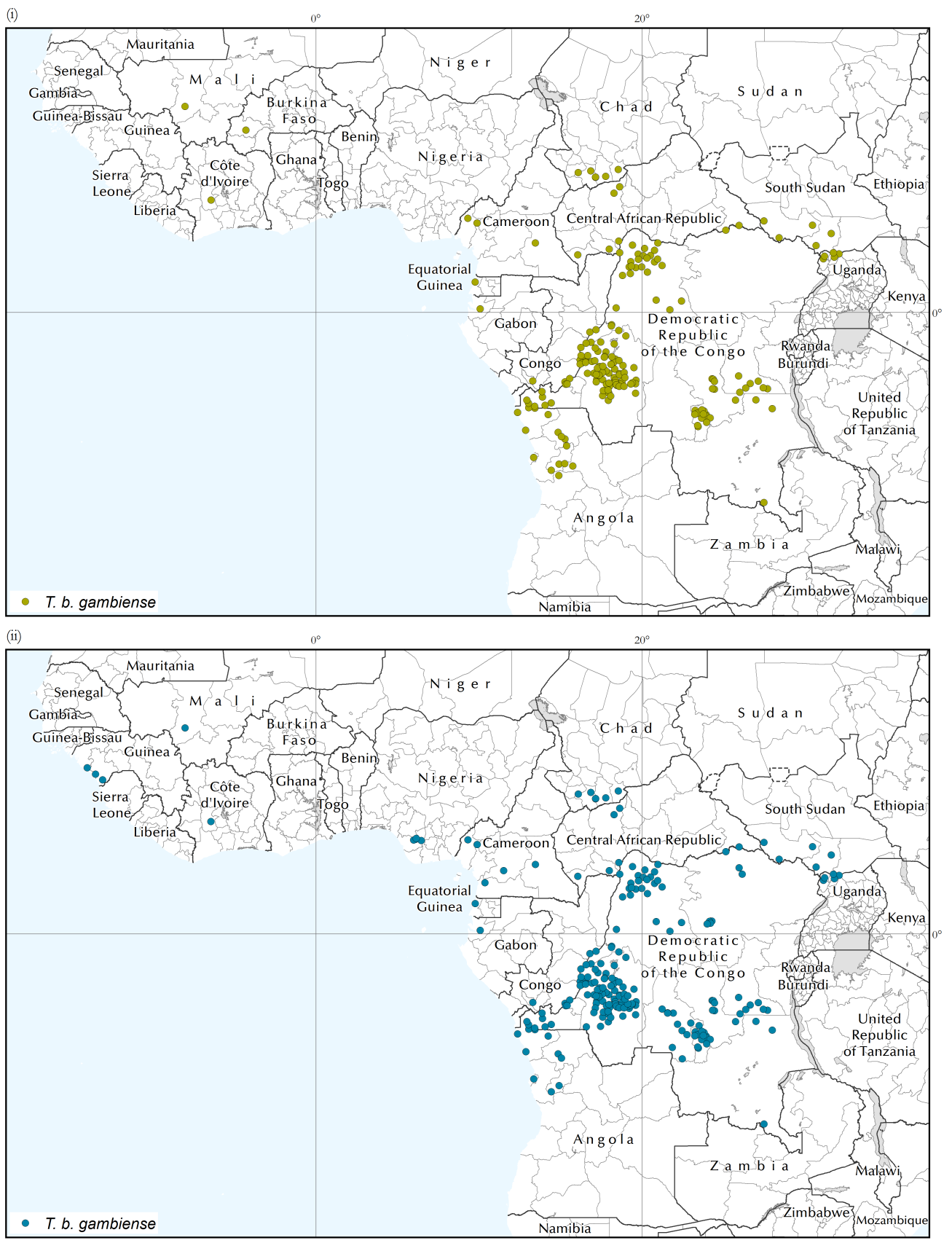


Fig D Geographic distribution of fixed health facilities having capacities for treatment of gambiense HAT second-stage infections with eflornithine (i) and with nifurtimox-eflornithine combination therapy (ii)
