# Supplementary material for: Monitoring the elimination of human African trypanosomiasis: Update to 2014
Source: PLoS Negl Trop Dis. 2017 May 22;11(5):e0005585. doi: 10.1371/journal.pntd.0005585 (PMC5456402; doi:10.1371/journal.pntd.0005585)
Supplement: S2 Text — People at risk of HAT that are potentially covered by facilities with diagnostic and treatment capabilities for HAT Table A. People at risk of Gambiense HAT that are potentially covered by facilities with diagnostic capabilities (2016, 2013 and difference 2016–2013) Table B. People at risk of Gambiense HAT that are potentially covered by facilities with treatment capabilities (2016, 2013 and difference 2016–2013) Table C. People at risk of Rhodesiense HAT that are potentially covered by facilities with diagnostic capabilities (2016) Table D. People at risk of Rhodesiense HAT that are potentially covered by facilities with treatment capabilities (2016). (DOCX) [file pntd.0005585.s002.docx]

# Additional file 2

## People at risk of HAT that are potentially covered by facilities with diagnostic and treatment capabilities for HAT

### Table A Gambiense HAT – Diagnosis

| **2016** |  |  |  |  |  |  |  |  |  |
| --- | --- | --- | --- | --- | --- | --- | --- | --- | --- |
| **Type of diagnosis** | **at-risk population potentially covered [%]** | | | | | | | | |
|  | ≤ 1 hour travel time | | | ≤ 3 hour travel time | | | ≤ 5 hour travel time | | |
|  | VH-H | M | L-VL | VH-H | M | L-VL | VH-H | M | L-VL |
| **Dx** | 45 | 43 | 53 | 83 | 75 | 78 | 92 | 88 | 88 |
| **DxC** | 45 | 43 | 52 | 83 | 75 | 77 | 92 | 88 | 88 |
| **DxS** | 39 | 38 | 50 | 79 | 71 | 71 | 91 | 86 | 86 |
| **DxP** | 36 | 34 | 44 | 78 | 69 | 71 | 90 | 85 | 84 |
| **DxPh** | 36 | 34 | 42 | 78 | 69 | 71 | 90 | 85 | 84 |

| **2013** |  |  |  |  |  |  |  |  |  |
| --- | --- | --- | --- | --- | --- | --- | --- | --- | --- |
| **Type of diagnosis** | **at-risk population potentially covered [%]** | | | | | | | | |
|  | ≤ 1 hour travel time | | | ≤ 3 hour travel time | | | ≤ 5 hour travel time | | |
|  | VH-H | M | L-VL | VH-H | M | L-VL | VH-H | M | L-VL |
| **Dx** | 44 | 44 | 42 | 78 | 75 | 74 | 89 | 86 | 86 |
| **DxC** | 44 | 44 | 42 | 78 | 75 | 74 | 89 | 86 | 86 |
| **DxS** | 33 | 39 | 40 | 71 | 72 | 70 | 86 | 85 | 83 |
| **DxP** | 32 | 39 | 38 | 71 | 71 | 69 | 85 | 84 | 82 |
| **DxPh** | 32 | 38 | 36 | 69 | 69 | 68 | 85 | 85 | 82 |

| **Δ (2016 – 2013)** |  |  |  |  |  |  |  |  |  |
| --- | --- | --- | --- | --- | --- | --- | --- | --- | --- |
| **Type of diagnosis** | **at-risk population potentially covered [%]** | | | | | | | | |
|  | ≤ 1 hour travel time | | | ≤ 3 hour travel time | | | ≤ 5 hour travel time | | |
|  | VH-H | M | L-VL | VH-H | M | L-VL | VH-H | M | L-VL |
| **Dx** | +0.9 | -0.4 | +10.9 | +5.0 | +0.2 | +3.7 | +2.9 | +1.8 | +2.3 |
| **DxC** | +0.9 | -0.5 | +9.5 | +5.0 | +0.2 | +3.5 | +2.9 | +1.7 | +2.2 |
| **DxS** | +5.7 | -1.6 | +10.4 | +8.0 | -0.6 | +1.1 | +4.7 | +1.5 | +3.0 |
| **DxP** | +3.8 | -4.8 | +5.5 | +7.7 | -1.9 | +2.1 | +4.8 | +0.7 | +1.9 |
| **DxPh** | +4.1 | -4.2 | +5.3 | +8.8 | -0.5 | +2.3 | +5.2 | +0.0 | +2.2 |

| **Dx**: any type of diagnosis  **DxC**: clinical diagnosis  **DxS**: serological diagnosis  **DxP**: parasitological diagnosis  **DxPh**: disease staging | **VH**: Very high risk  **H**: High risk  **M**: Moderate risk  **L**: Low risk  **VL**: Very low risk |
| --- | --- |

### Table B Gambiense HAT – Treatment

| **2016** |  |  |  |  |  |  |  |  |  |
| --- | --- | --- | --- | --- | --- | --- | --- | --- | --- |
| **Type of treatment** | **at-risk population potentially covered [%]** | | | | | | | | |
|  | ≤ 1 hour travel time | | | ≤ 3 hour travel time | | | ≤ 5 hour travel time | | |
|  | VH-H | M | L-VL | VH-H | M | L-VL | VH-H | M | L-VL |
| **Tx** | 44 | 41 | 42 | 83 | 73 | 73 | 92 | 87 | 85 |
| **Tx1P** | 44 | 41 | 42 | 83 | 73 | 73 | 92 | 87 | 85 |
| **Tx2M** | 22 | 28 | 29 | 49 | 58 | 55 | 62 | 73 | 67 |
| **Tx2E** | 22 | 29 | 37 | 48 | 60 | 65 | 61 | 76 | 76 |
| **Tx2N** | 35 | 32 | 39 | 77 | 68 | 70 | 89 | 84 | 83 |

| **2013** |  |  |  |  |  |  |  |  |  |
| --- | --- | --- | --- | --- | --- | --- | --- | --- | --- |
| **Type of treatment** | **at-risk population potentially covered [%]** | | | | | | | | |
|  | ≤ 1 hour travel time | | | ≤ 3 hour travel time | | | ≤ 5 hour travel time | | |
|  | VH-H | M | L-VL | VH-H | M | L-VL | VH-H | M | L-VL |
| **Tx** | 44 | 42 | 38 | 78 | 74 | 71 | 89 | 86 | 84 |
| **Tx1P** | 44 | 42 | 38 | 78 | 74 | 71 | 89 | 86 | 84 |
| **Tx2M** | 29 | 36 | 33 | 68 | 69 | 67 | 84 | 83 | 81 |
| **Tx2E** | 29 | 36 | 33 | 68 | 70 | 66 | 84 | 83 | 79 |
| **Tx2N** | 29 | 32 | 31 | 67 | 64 | 63 | 84 | 76 | 76 |

| **Δ (2016 – 2013)** |  |  |  |  |  |  |  |  |  |
| --- | --- | --- | --- | --- | --- | --- | --- | --- | --- |
| **Type of treatment** | **at-risk population potentially covered [%]** | | | | | | | | |
|  | ≤ 1 hour travel time | | | ≤ 3 hour travel time | | | ≤ 5 hour travel time | | |
|  | VH-H | M | L-VL | VH-H | M | L-VL | VH-H | M | L-VL |
| **Tx** | +0.6 | -1.6 | +4.1 | +4.9 | -0.5 | +2.1 | +2.9 | +1.4 | +1.4 |
| **Tx1P** | +0.6 | -1.6 | +4.1 | +4.9 | -0.5 | +2.1 | +2.9 | +1.4 | +1.4 |
| **Tx2M** | -6.9 | -8.9 | -4.7 | -18.5 | -11.6 | -11.6 | -22.2 | -10.0 | -14.3 |
| **Tx2E** | -7.7 | -7.3 | +3.8 | -19.7 | -9.4 | -1.0 | -22.6 | +7.4 | -3.7 |
| **Tx2N** | +5.5 | 0.0 | +8.4 | +9.7 | +4.0 | +7.5 | +5.7 | +7.8 | +6.9 |

| **Tx**: any type of treatment  **Tx1P**: treatment of first-stage infection with pentamidine  **Tx2M**: treatment of second-stage infection with melarsoprol  **Tx2E**: treatment of second-stage infection with eflornithine  **Tx2N**: treatment of second-stage infection with nifurtimox-eflornithine combination therapy (NECT) | **VH**: Very high risk  **H**: High risk  **M**: Moderate risk  **L**: Low risk  **VL**: Very low risk |
| --- | --- |

### Table C Rhodesiense HAT – Diagnosis

| **2016** |  |  |  |  |  |  |  |  |  |
| --- | --- | --- | --- | --- | --- | --- | --- | --- | --- |
| **Type of diagnosis** | **at-risk population potentially covered [%]** | | | | | | | | |
|  | ≤ 1 hour travel time | | | ≤ 3 hour travel time | | | ≤ 5 hour travel time | | |
|  | VH-H | M | L-VL | VH-H | M | L-VL | VH-H | M | L-VL |
| **Dx** | - | 61 | 40 | - | 88 | 74 | - | 97 | 85 |
| **DxC** | - | 61 | 40 | - | 88 | 74 | - | 97 | 85 |
| **DxP** | - | 61 | 35 | - | 88 | 72 | - | 97 | 84 |
| **DxPh** | - | 54 | 33 | - | 82 | 72 | - | 94 | 83 |

| **Dx**: any type of diagnosis  **DxC**: clinical diagnosis  **DxP**: parasitological diagnosis  **DxPh**: disease staging | **VH**: Very high risk  **H**: High risk  **M**: Moderate risk  **L**: Low risk  **VL**: Very low risk |
| --- | --- |

### Table D Rhodesiense HAT – Treatment

| **2016** |  |  |  |  |  |  |  |  |  |
| --- | --- | --- | --- | --- | --- | --- | --- | --- | --- |
| **Type of treatment** | **at-risk population potentially covered [%]** | | | | | | | | |
|  | ≤ 1 hour travel time | | | ≤ 3 hour travel time | | | ≤ 5 hour travel time | | |
|  | VH-H | M | L-VL | VH-H | M | L-VL | VH-H | M | L-VL |
| **Tx** | - | 54 | 33 | - | 82 | 72 | - | 94 | 83 |
| **Tx1S** | - | 54 | 33 | - | 82 | 72 | - | 94 | 83 |
| **Tx2M** | - | 54 | 33 | - | 82 | 72 | - | 94 | 83 |

| **Tx**: any type of treatment  **Tx1S**: treatment of first-stage infection with suramin  **Tx2M**: treatment of second-stage infection with melarsoprol | **VH**: Very high risk  **H**: High risk  **M**: Moderate risk  **L**: Low risk  **VL**: Very low risk |
| --- | --- |
